# The effects of individual status and group performance on network ties among teammates in the National Basketball Association

**DOI:** 10.1371/journal.pone.0196013

**Published:** 2018-04-30

**Authors:** Jeremy Koster, Brandy Aven

**Affiliations:** 1 Department of Anthropology, University of Cincinnati, Cincinnati, OH, United States of America; 2 Department of Human Behavior, Ecology, and Culture, Max Planck Institute for Evolutionary Anthropology, Leipzig, Germany; 3 Tepper School of Business, Carnegie Mellon University, Pittsburgh, PA, United States of America; Centre National de la Recherche Scientifique, FRANCE

## Abstract

For individuals, status is derived both from their personal attributes and the groups with whom they are affiliated. Depending on the performance of their groups, the status of individuals may benefit or suffer from identifying closely with the group. When the group excels, high-status members potentially receive much of the credit and increased status. Conversely, high-status members of underperforming groups potentially suffer disproportionate declines in their status relative to the low-status group members. We therefore predict an interaction between group performance and individual status on the willingness to associate with the group and its members. We test our prediction by examining social media ties among teammates in the National Basketball Association. Specifically, we investigate the “following” ties of teammates on Twitter at the end of the 2014–2015 season. Elections to All-Star games are used to measure the status of players, and team performance is measured by recent success in the postseason playoffs. The results show that compared to high-status players on successful teams, high-status players on underperforming teams are less likely to follow their teammates. This result aligns with research on status inconsistency, suggesting that individuals deemphasize their group affiliation when it jeopardizes their individual status. An additional contribution is the advancement of the probit Social Relations Model for the analysis of binary ties in social networks.

## Introduction

Status is a position in a social hierarchy that is based on social esteem and respect, which are largely demonstrated through deference [1, 2]. Status is derived not only from individuals’ past performances and comparisons to others [3–5], but also from variation in the performance of the groups to which individuals are affiliated [6, 7]. Individuals therefore can alter their individual status through their associations to high or low performing groups [8, 9], and the status of individuals and the performance of their groups combine to exhibit effects on subsequent career outcomes [3, 10, 11]. Given these effects, additional research is needed on the strategies that individuals employ when their status is inconsistent with their current group’s performance. Extending scholarship on expectation states theory and status inconsistency [12, 13], this study examines the interactive effects of status and group performance on the willingness of individuals to associate and identify with their groups.

In settings where the quality of individuals is difficult to discern, their status often serves as an imperfect proxy for quality [4, 14]. Purported quality is expected to vary with status, and high status can positively bias evaluators (e.g., leading them to discount evidence of subpar quality) [12, 13, 15]. However, associations with underperforming groups can potentially jeopardize individuals’ status, particularly for those with high status [16, 17]. In labor markets, for example, individuals who are strongly associated with failed organizations suffer relatively greater reductions in status and career prospects [11]. To mitigate these effects, individuals may distance themselves from other group members to deemphasize their affiliation with the underperforming group [18, 19].

Professional sports provide an ideal opportunity to investigate the effects of individuals’ status and heterogeneous group performance on the behavior of players toward their teammates. Notably, high-status players are disproportionately responsible for their teams’ performance because they typically receive more playing time and more opportunities to impact the outcomes of games. In sports that require considerable coordination among players, such as basketball, talented players are also expected to elevate the performance of their teammates [20]. In discussions about the greatest players in the history of the sport, journalists frequently assign considerable importance to the number of league championships that elite players have won with their teams [21–24]. Conversely, high-status players on underperforming teams are allegedly to blame for not leading their teammates to victory [25, 26]. The status of elite players is therefore expected to be especially affected by variation in team performance. When their teams are chronically unsuccessful, the withdrawal of high-status players from team networks is potentially interpretable as a strategic attempt to retain status while implicitly redirecting culpability for the subpar organizational performance.

Given these considerations, we hypothesize that team performance moderates the propensity for high-status players to associate with their teammates. On winning teams, high-status players primarily realize further enhancements to their status, and they potentially signal effective leadership and high cooperation by associating with other team members [27]. By contrast, the subpar performance of losing teams potentially threatens the status of elite players, leading them to distance themselves from teammates. Statistically, these tendencies will result in an interaction of player status and team performance on the willingness of players to associate and identify with their teammates. For high-status players, in other words, the propensity for affiliating with teammates is expected to be particularly responsive to variation in team performance.

## Methods

### Research setting

To test our hypothesis, we examined the social media ties of teammates on professional basketball teams in the National Basketball Association (NBA). Specifically, we investigated the following ties of teammates on Twitter, a popular social media service that allows users to post and exchange messages from computers and mobile devices. One mechanism for interacting on Twitter is to “follow” another user, which allows followers to read the messages posted by the users they follow. It is possible for following ties to be unreciprocated because upon being followed by someone, Twitter users can either choose to follow or not follow the alter’s Twitter account.

Since its inception in 2006, Twitter has become a popular platform for professional athletes to engage with teammates, fans, and members of the media [28]. All-Star players frequently garner millions of followers on Twitter, providing opportunities for athletes to curate a public image and brand that can be strategically leveraged into lucrative endorsements [29]. Players also use their Twitter accounts to motivate or criticize their teammates, and journalists closely monitor players’ accounts for signs of dissension on teams [30]. Although there are potentially multiple motivations to follow a teammate’s Twitter account, a tie is interpretable as a low-cost means of demonstrating an affiliation [5]. Given that initiating a following tie on Twitter requires little more than a few seconds, the low cost of tie formation accentuates the import of decisions not to follow specific teammates.

To generate our sample, we identified the NBA players who maintained a Twitter account by reviewing the internet profiles of active players from a company that aggregates biographical information about players [31]. We excluded player’s non-personal accounts, which primarily served to promote and market players’ special interests, such as charities or summer camps associated with the players. On average, each NBA team had an average of 11 current players with Twitter accounts (SD = 1.39), as compared to roster sizes of approximately 14 players. The sample of 330 individuals therefore includes about 79% of the players on league rosters at the time. Using NodeXL software [32], we then downloaded the following ties of players to their teammates. The download occurred on May 28, 2015, on the eve of the final playoff series to decide the league’s champion. The sample of team networks therefore represents the status of following ties among current members of the teams near the end of the 2014–2015 season. All data collection complied with the terms of service of Twitter.

**Fig 1:**
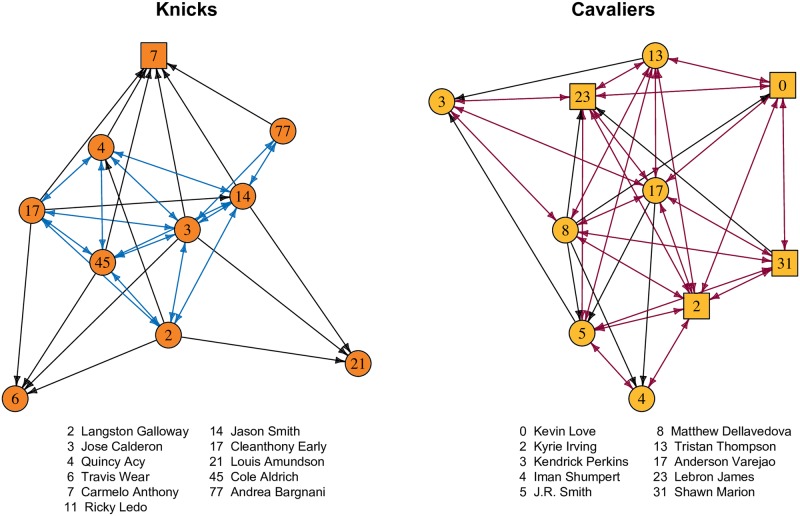
Twitter networks of the New York Knicks and the Cleveland Cavaliers. Colored edges indicate reciprocated ties. Black edges denote unreciprocated ties. Square nodes denote players who were selected for at least one All-Star game as of May, 2015. Round nodes indicate players who had not been selected for an All-Star game.

For general insight into the data structure of the teams’ Twitter networks, Fig 1 presents visualizations of the network for two teams, the New York Knicks and the Cleveland Cavaliers. These teams were selected as examples because their respective leading scorers, Carmelo Anthony and Lebron James, had comparable status as individuals while playing for teams with divergent success. For example, the performance of these teams during the 2014–2015 season differed dramatically, as the Knicks had the worst winning percentage in their franchise’s history whereas the Cavaliers were the league’s runner-up after advancing to the playoff’s final round. The Twitter networks of the teams moderately vary in their density, as only 41% of the possible following ties appear among the Knicks whereas the density of the Cavaliers network is 68%. Among the Cavaliers, there are no players who do not follow at least one of their teammates, whereas three players on the Knicks do not follow any of their teammates, a group that includes Carmelo Anthony.

### Analysis

Our dependent variable is whether player *i* follows the Twitter account of teammate *j* on team *k*. The variable is therefore dyadic, and the repeated observations of individuals as players and teammates in the dataset introduce structural dependencies that are well known to scholars of social network analysis [33]. To analyze these data, we therefore use an adaptation of the multilevel Social Relations Model (SRM) for binary outcomes [34]. As in conventional applications of the SRM for continuous responses [35, 36], the objective of the probit SRM is to partition the variance of the outcome variable as a function of heterogeneity in actors (i.e., the players on Twitter), partners (i.e., the teammates whom the players can follow), and dyadic effects that relate to the penchant for reciprocity in social networks. Using a probit link function, we formulate the model as a latent-response model. Thus, underlying the observed binary response, *y*_*ik*, *jk*_, we imagine that there is an unobserved or latent continuous response, $y_{ik,jk}^{*}$, representing the propensity for a tie. If this latent response is 0 or greater, then the observed response is 1; otherwise, the observed response is 0:

$$\begin{array}{c} y_{ik,jk}=\{\begin{array}{cc} 1, & y_{ik,jk}^{*}\geq 0\\ 0, & y_{ik,jk}^{*}<0 \end{array}\; \end{array}$$

A linear regression SRM is then specified for the latent response, $y_{ik,jk}^{*}$

$$\begin{array}{c} y_{ik,jk}^{*}=x_{ik,jk}^{\prime }\beta +m_{k}+a_{ik}+b_{jk}+e_{ik,jk} \end{array}$$

where $x_{ik,jk}^{\prime }$ denotes the vector of covariates and *β* the associated vector of regression coefficients, *m*_*k*_ is a random effect for team *k*, *a*_*ik*_ and *b*_*jk*_ denote random effects for player *i* and teammate *j*, respectively, and *e*_*ik*, *jk*_ denotes the directed dyadic random effect. The variance and covariance structure of the model is notated as follows:

$$m_{k}∼\text{Normal}\;\left(0,\sigma_{m}^{2}\right)$$

$$\left(\begin{array}{c} a_{ik}\\ b_{ik} \end{array}\right)∼\text{MVNormal}\;\left\{\left(\begin{array}{c} 0\\ 0 \end{array}\right),\left(\begin{array}{cc} \sigma_{a}^{2} & \\ \sigma_{ab} & \sigma_{b}^{2} \end{array}\right)\right\},\;\;\rho_{ab}=\frac{\sigma_{ab}}{\sqrt[{}]{\sigma_{a}^{2}}\sqrt[{}]{\sigma_{b}^{2}}}$$

$$\left(\begin{array}{c} e_{ik,jk}\\ e_{jk,ik} \end{array}\right)∼\text{MVNormal}\;\left\{\left(\begin{array}{c} 0\\ 0 \end{array}\right),\left(\begin{array}{cc} 1 & \\ \sigma_{ee} & 1 \end{array}\right)\right\},\;\;\rho_{ee}=\sigma_{ee}$$

The group-level random effect, *m*_*k*_, reflects the extent to which binary ties between members of group *k* are more or less prevalent than the average group. The effect for players, *a*_*ik*_, captures the extent to which an individual player differs from average in terms of directing following ties toward teammates. Likewise, the effect for teammates, *b*_*jk*_, reflects a comparable deviation from average for teammates in terms of attracting following ties on Twitter. The correlation between these respective effects, *ρ*_*ab*_, is known as the generalized reciprocity correlation [37]. Note that “generalized reciprocity” is not to be confused with alternative connotations of the terminology that are common to Social Exchange Theory [38].

The model also includes directed effects, *e*_*ij*_ and *e*_*ji*_, which are essentially the residuals of the model. To permit identification of the probit model, the variances of these effects are constrained to 1. The correlation between these effects, *ρ*_*ee*_, is conventionally known as the dyadic reciprocity correlation. When the correlation is positive, it indicates that a tie from player *i* to teammate *j* tends to be reciprocated, adjusting for their respective tendencies as directors and recipients of following ties. Although negative correlations are also possible, our expectation conforms to sociological insights that predict positive dyadic reciprocity between teammates [39].

The relative importance of team, player, teammate, and dyadic effects as sources of variation can be summarized by dividing each estimated variance by the total of the four estimated variances.

$$\begin{array}{c} p_{m}=\frac{\sigma_{m}^{2}}{\sigma_{m}^{2}+\sigma_{a}^{2}+\sigma_{b}^{2}+1} \end{array}$$

$$\begin{array}{c} p_{a}=\frac{\sigma_{a}^{2}}{\sigma_{m}^{2}+\sigma_{a}^{2}+\sigma_{b}^{2}+1} \end{array}$$

$$\begin{array}{c} p_{b}=\frac{\sigma_{b}^{2}}{\sigma_{m}^{2}+\sigma_{a}^{2}+\sigma_{b}^{2}+1} \end{array}$$

$$\begin{array}{c} p_{e}=\frac{1}{\sigma_{m}^{2}+\sigma_{a}^{2}+\sigma_{b}^{2}+1} \end{array}$$

These statistics are referred to in the multilevel modeling literature as variance partition coefficients, or VPCs [40].

### Predictor variables

As predictors of Twitter ties among teammates, we collected publicly available information on all of the teams and their players, including the teams’ recent performance and players’ league tenure, salaries, Twitter account tenure, height, college alma mater, and appearances in the league’s All-Star games. With the exception of account tenure (see below), all data were obtained from basketball-reference.com [31]. We also sought to include data on the racial self-identification of the NBA players; however, although such data on players’ races are collected by the NBA in partnership with the Institute for Diversity and Ethics In Sport, these data are not publicly available [41]. Descriptive statistics for all predictor variables used in this analysis are included in Table 1.

**Table 1:** Variable names, descriptions, and summary statistics for the possible directed Twitter following ties among NBA teammates (*n* = 3,356).

*Variable*	*Description*	*Mean*	*Std. Dev.*	*Min*	*Max*
*Teams (n = 30)*					
Team Playoff Performance^a^	Weighted average of annual playoff victories, 2007–2015, square root transformed	1.453	0.759	0	2.864
*Individuals (n = 330)*					
All-Star Appearances^b^	The number of All-Star games in which the player has participated	0.649	2.071	0	17
League Tenure (log)^a^	Log transformed number of seasons in which the player has been active	1.457	0.829	0	2.944
Salary (log)^a^	Player’s log transformed salary, measured in millions of dollars	0.978	1.217	−3.524	3.157
Time Using Twitter^a^	Age of the player’s Twitter account, measured in years	4.528	1.392	0.066	6.727
Height^a^	Player ’s height, measured in inches	79.16	3.4	71	86
*Dyads (n = 1678)*					
Years as Teammates^b^	The number of seasons in which players *i* and *j* have played for the same team	1.563	1.097	0	13
Same College	A binary variable, coded as 1 when *i* and *j* attended the same college or university	0.016		0	1

^a^ Denotes variables that were *z*-score standardized prior to the analysis, relative to the means and standard deviations in this table.

^b^ Denotes variables that were proportionally standardized prior to the analysis, relative to the maximum value in this table.

#### All-Star appearances

As a measure of individual status among NBA players, we use the number of appearances in the league’s All-Star game. Participation in All-Star games is a common measure of status in research on professional athletes [15, 42, 43]. During the timeframe encompassed by this study, the starting players in the All-Star game were selected via voting by fans whereas the reserves on the teams were chosen by the league’s head coaches. As in research on All-Stars in Major League Baseball, we use the cumulative number of All-Star appearances because we expect players’ status to increase incrementally with each appearance [15, 43]. To facilitate estimation of models, this variable is transformed proportionally by dividing each player’s number of All-Star appearances by the maximum number of appearances (17, by Kobe Bryant). We refer to players without any All-Star nominations as “non-All-Star players”.

#### Team playoff performance

As a measure of team performance, we consider the number of playoff games won by team *k* between 2007 and 2015. As an alternative to performance during the regular season, success in the playoffs is used in this analysis because postseason games receive considerably more visibility and media attention, thus providing key opportunities for players and teams to boost their status [44, 45]. As a starting point, the 2007 NBA playoffs were selected because this was the first season following Twitter’s debut. It may be anticipated, though, that more recent team performance is especially salient for players. We therefore create a weighted average of playoff wins per season for each team, with weights inversely related to the number of years preceding the Twitter download in 2015. For example, wins in 2007 have approximately 11% of the weight of wins in 2015.

This weighted average is positively skewed, so first a square root transformation is applied and then subsequently the transformed variable is *z*-score standardized to facilitate estimation. The interaction of this variable, *team playoff performance*, with the *All-Star appearances* of player *i* is used to test our hypothesis of the relationship between player status and team performance.

#### Player-level covariates

We collected data on other player-level attributes. Importantly, we control for the players’ *league tenure*, which accounts for the fact that the number of possible All-Star appearances depends largely on the number of seasons that the player has been in the league. This variable is log-transformed to account for its positive skew.

We also include data on the *salary* of individuals *i* and *j*. Notably, salaries in the NBA are governed by a “salary cap” that limits the range of teams’ spending on contracts while simultaneously restricting the potential earnings of inexperienced players and the maximum value of permissible contracts [46]. These considerations result in an imperfect correlation between the performance of players and the value of their contracts, particularly as the exact range of the salary cap is adjusted over time.

Players vary in their affinity for using Twitter, and early adopters of the service plausibly exhibit greater propensity for following other users. Similarly, players who have long maintained Twitter accounts have potentially had more opportunities to accumulate following ties from their teammates. The ages of players’ accounts were determined via searches on the website, Tw Birthday (twbirthday.com), which draws user information directly from Twitter. This variable is hereafter described as *time using Twitter*.

Finally, we include data on the players’ *height*, which in other contexts has been a predictor of social status and leadership [47]. The NBA is an unusual context, however, because in terms of positions, point guards are often regarded as team leaders despite being among the shortest players [48]. Furthermore, whereas some of the game’s most decorated players have been very tall, some players have lengthy careers largely because of their size, not because they are viewed as talented players [49]. In models that account for league tenure and All-Star appearances, we therefore anticipate that taller players receive fewer following ties.

For all player-level variables, the model includes the respective values for both player *i* and teammate *j*. To facilitate estimation and interpretation of models, the aforementioned variables were all *z*-score standardized relative to their means and standard deviations in Table 1.

#### Dyad-level covariates

Including the extensive travel schedules, teammates have substantial opportunities to socialize and interact throughout a season, which we expect to result in the formation of ties on Twitter. By charting participation in games during the regular season, we created a variable that accounts for any season in which two players were active members of the same team. We then summed this variable to generate a variable of overlapping team membership, *years as teammates*. This variable was transformed proportionally by dividing each dyad’s value by the maximum number of years in which two players were teammates (13, by Tony Parker and Manu Ginobli). The operationalization of this variable does not presume that the players were concurrently on the playing roster throughout the season.

In some cases, teammates on NBA teams attended the same college or university. A binary variable, *same college*, was generated to denote dyads in which player *i* and teammate *j* share a college-level affiliation. This variable does not presume that either player graduated, nor that they played simultaneously as teammates for the college. However, players who attended the same college sometimes end up interacting during summertime workouts that are held informally on campus. Combined with the social identification that stems from their organizational affiliation [50], we therefore expect that this variable will predict increases in Twitter ties among NBA teammates from the same college.

### Modeling strategy and estimation

We estimate four models. The first model is an “empty” model that includes only the intercept and the random effects, which reveals the structure of the dataset and the relative importance of the VPCs. Second, we fit a model that includes the interaction of player *i*’s status and the measure of team performance because our hypothesis predicts that All-Star players on successful teams are likely to follow teammates whereas All-Star players on underperforming teams will distance themselves from teammates. Our third model considers the effect of teammate *j*’s status as an alter, which potentially moderates the relationship between player *i*’s status and team performance. This model therefore includes a three-way-interaction, and to mitigate concerns about the misinterpretation of interaction terms in nonlinear models [51], we interpret our models primarily via plots of model predictions [52].

Our fourth model includes covariates and interactions that potentially provide alternative explanations to the effects of status and team performance. For instance, successful teams might exhibit less roster turnover than underperforming teams, and it is therefore important to control for the amount of time that teammates have played together. We particularly aim to account for the effects of homophily, the propensity for individuals to form network ties with similar others [39, 53]. Therefore, we create dyad-level covariates by interacting the main effects of the player-level variables [34]. For example, if players preferentially affiliate and follow teammates with similar incomes, that assortment would be evident in the multiplicative interaction term of *salary* for player *i* and teammate *j*.

We fit our models using Markov chain Monte Carlo (MCMC) methods, as implemented in the Stat-JR software environment [54]. We specify uninformative prior distributions for all parameters. Estimating four parallel chains, we specify a burn-in of 100,000 iterations, after which we sample an addition 1 million iterations with a “thinning” parameter of 200 iterations (so that a total of 5,000 samples are stored from each chain). In our results, we present the means and standard deviations of these stored samples. Standard MCMC diagnostics suggest that the models mixed well and that all chains converged to the same distribution. All reported parameters had effective sample sizes of at least 8,000 posterior samples. For replicative purposes, the raw data and Stat-JR template are included as supplemental files.

## Results

Model results are presented in Table 2.

**Table 2:** Probit SRM results. Reported parameters are the means and standard deviations (in parentheses) from the posterior samples. Note that all continuous variables have been standardized as either proportions or *z*-scores. Asterisks between terms denote interaction effects. Confidence in model coefficients can be inferred by dividing the posterior means by the posterior standard deviations (and potentially by comparing the quotients against a standard normal distribution).

Parameter		Model 1	Model 2	Model 3	Model 4
*β*_0_	Intercept	0.09 (0.13)	0.20 (0.13)	0.10 (0.13)	−0.85 (0.17)
*β*_1_	All-Star Appearances (*i*)		−2.92 (0.62)	−1.95 (0.82)	−2.16 (0.93)
*β*_2_	Team Playoff Performance (*k*)		0.23 (0.13)	0.15 (0.13)	0.19 (0.15)
*β*_3_	All-Star Appearances (*i*) *		1.80 (0.64)	2.15 (0.86)	2.11 (0.93)
	Team Playoff Performance (*k*)				
*β*_4_	All-Star Appearances (*j*)			1.17 (0.65)	0.42 (0.64)
*β*_5_	All-Star Appearances (*i*) *			−1.23 (5.25)	−1.37 (6.13)
	All-Star Appearances (*j*)				
*β*_6_	All-Star Appearances (*j*) *			0.41 (0.69)	0.07 (0.66)
	Team Playoff Performance (*k*)				
*β*_7_	All-Star Appearances (*i*) *			4.54 (6.97)	1.59 (7.82)
	All-Star Appearances (*j*) *				
	Team Playoff Performance (*k*)				
*β*_8_	League Tenure (*i*)				−0.27 (0.09)
*β*_9_	League Tenure (*j*)				−0.17 (0.06)
*β*_10_	League Tenure (*i*) *				0.12 (0.04)
	League Tenure (*j*)				
*β*_11_	Salary (*i*)				0.16 (0.09)
*β*_12_	Salary (*j*)				0.33 (0.06)
*β*_13_	Salary (*i*) *				0.06 (0.04)
	Salary (*j*)				
*β*_14_	Time Using Twitter (*i*)				0.17 (0.08)
*β*_15_	Time Using Twitter (*j*)				0.23 (0.05)
*β*_16_	Time Using Twitter (*i*) *				0.04 (0.04)
	Time Using Twitter (*j*)				
*β*_17_	Height (*i*)				0.09 (0.07)
*β*_18_	Height (*j*)				0.04 (0.05)
*β*_19_	Height (*i*) *				−0.02 (0.04)
	Height (*j*)				
*β*_20_	Years as Teammates				8.67 (0.87)
*β*_21_	Same College				0.78 (0.31)
$\sigma_{{}_{m}^{2}}$	Team-level Variance	0.15 (0.15)	0.13 (0.14)	0.14 (0.14)	0.22 (0.18)
$\sigma_{{}_{a}^{2}}$	Player-level Variance	1.17 (0.15)	1.18 (0.15)	1.17 (0.15)	1.29 (0.17)
$\sigma_{{}_{b}^{2}}$	Teammate-level Variance	0.71 (0.10)	0.71 (0.10)	0.68 (0.10)	0.50 (0.08)
*ρ*_*ab*_	Generalized Reciprocity	0.75 (0.04)	0.78 (0.03)	0.78 (0.03)	0.80 (0.04)
*ρ*_*ee*_	Dyadic Reciprocity	0.90 (0.03)	0.90 (0.03)	0.90 (0.03)	0.88 (0.03)
*p*_*m*_	Team-level VPC	0.05 (0.05)	0.04 (0.04)	0.04 (0.04)	0.07 (0.05)
*p*_*a*_	Player-level VPC	0.39 (0.03)	0.39 (0.03)	0.39 (0.03)	0.43 (0.04)
*p*_*b*_	Teammate-level VPC	0.23 (0.02)	0.23 (0.02)	0.23 (0.02)	0.17 (0.02)
*p*_*e*_	Dyad-level VPC	0.33 (0.03)	0.33 (0.03)	0.34 (0.03)	0.34 (0.03)

### Model 1: The “empty” model

In the model that includes only the intercept and the random effects, there is relatively little variation that is attributable to team-level differences (*p*_*m*_ = 0.05). By contrast, the model indicates that players vary considerably in their propensity for following teammates on Twitter (*p*_*a*_ = 0.39). There is also substantial heterogeneity in the extent to which players attract following ties (*p*_*b*_ = 0.23). The generalized reciprocity correlation is high (*ρ*_*ab*_ = 0.75), indicating that players who follow many teammates also tend to receive many following ties. In the parlance of social network analysis, this implies a positive correlation between in-degree centrality and out-degree centrality [55].

The dyadic variance is also high (*p*_*e*_ = 0.33), suggesting that dyadic attributes structure the formation of following ties among teammates. The dyadic reciprocity correlation is very high (*ρ*_*ee*_ = 0.90), which indicates that following ties are typically reciprocated. Furthermore, these results imply that unreciprocated ties often reflect attributes of the individuals, not the dyads. In other words, ties may be unreciprocated because a player seldom follows any teammates, including the individual who had followed him.

### Model 2: The interaction of player *i*’s status and team performance

Model 2 suggests that All-Star players on underperforming teams are less likely to follow their teammates on Twitter than their counterparts on successful teams. The predicted effect is substantial. As *team playoff performance* increases from one standard deviation below the mean to one standard deviation above the mean, the predicted probability for a following tie from a 4-time All-Star increases from 13% to 56%. For a non-All-Star player, the corresponding increases are more modest, increasing from a probability of 49% to 67% for following teammates as team performance improves.

Although this interaction effect provides support for our hypothesis, the effect in this model could be a by-product of homophilous affiliations among high-status individuals if All-Star players are disproportionately represented on successful teams. A supplemental analysis corroborates this possibility by indicating that players with All-Star experience are more common on high-performing teams (S1 Fig). The next model therefore considers the effects of teammate *j*’s status.

### Model 3: Moderating effects of teammate *j*’s status

**Fig 2:**
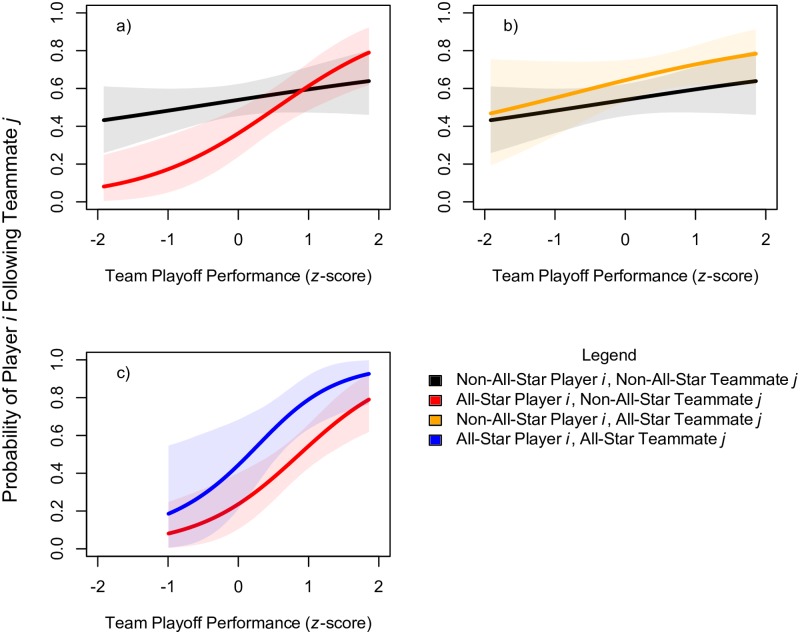
Predicted probabilities of following ties as a function of team performance and the All-Star status of player *i* and teammate *j*. All model predictions are based on simulations from the posterior samples of Model 3. All-Star players and teammates are assumed to have 4 All-Star appearances, and non-All-Star players are assumed to have none. Shaded intervals show the 90% confidence around model predictions. In panel c, the predictions are depicted across a reduced range to avoid out-of-sample predictions because although there were three teams whose playoff performance was less than one standard deviation below the mean, none of these teams had more than one player with experience as an All-Star.

Fig 2 plots the predictions of Model 3, which includes the three-way interactions of the respective statuses of player *i* and teammate *j* and the performance of their team. In this model, the hypothesized interaction of player *i*’s status and *team playoff performance* continues to be evident (Fig 2, panel a). In other words, the interaction effect is not simply a by-product of the greater abundance of All-Star players on successful teams. Overall, the results are consistent with the prediction that All-Star players on losing teams deemphasize their association and identification with the team by withdrawing or abstaining from intra-team connections.

The model provides modest support for a positive main effect of teammate *j*’s status (Fig 2, panel b). However, this effect is attenuated in models that include additional covariates, seemingly because variables such as teammate *j*’s salary are clearer predictors of incoming Twitter ties.

There is little evidence for heterogeneous affiliation among All-Star players across the range of team performance (Fig 2, panel c). For instance, the model predicts that All-Star teammates on losing teams are almost equally unlikely to attract following ties from their high-status teammates. In general, however, there are few dyads composed of two All-Stars, and there is concomitant uncertainty in the model parameters and predictions pertaining to these dyads.

### Model 4: Full model with covariates

The interaction effect of player status and team performance remain consistent in Model 4, which again shows that high-status players are less likely to follow their teammates on unsuccessful teams (S2 Fig). This result provides evidence that the effect is not a by-product of homophily or variation in experience as teammates.

**Fig 3:**
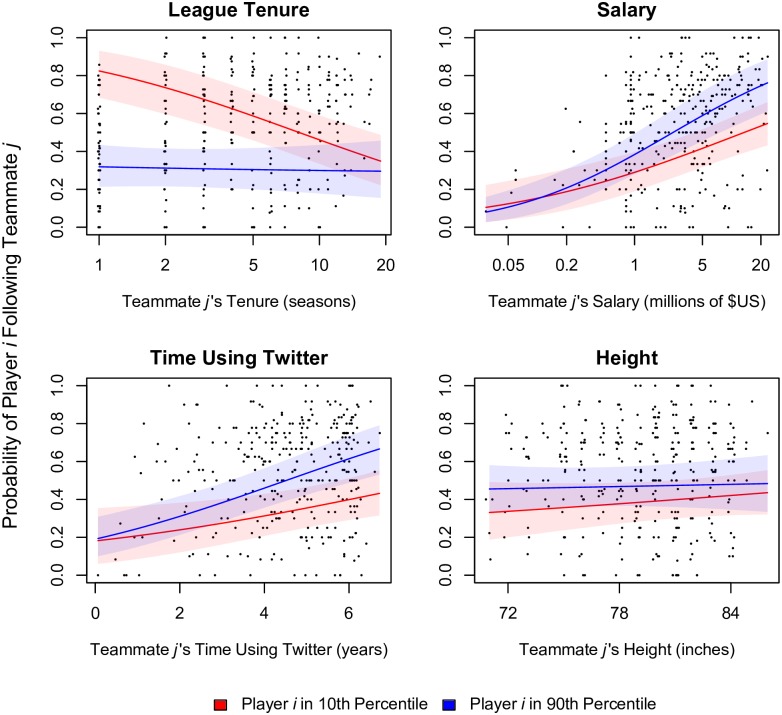
Predicted probabilities of following ties by the interaction of player-level covariates. Model predictions are based on simulations from the posterior samples of Model 4. Plots are oriented toward the perspective of teammate *j* as an alter, and the horizontal axis in each panel depicts the range of variation in the teammate-level covariates. To illustrate the interaction effects, two discrete values for player *i* are supplied. Red lines show the predicted probabilities when player *i* has a low value in the 10th percentile of the empirical distribution of the covariate corresponding to the respective panel. Blue lines depict predicted probabilities for high values (90th percentile) of the covariate. For example, red and blue lines in the first panel show predictions for inexperienced and veteran players, respectively. The predictions assume that both player *i* and teammate *j* have been teammates for one year and that neither has been previously recognized as an All-Star player. All other parameters are held constant at their means or reference values. Points show the proportion of incoming Twitter ties received by teammate *j*. Shaded intervals show the 90% confidence around model predictions.

Other parameters in the model merit brief attention. The predicted effects of the player-level covariates are depicted in Fig 3. In terms of *league tenure*, the model indicates that players with less experience follow more of their teammates, and they are particularly likely to follow comparably inexperienced players. Regarding *salary*, highly paid teammates attract relatively more Twitter followers, and highly paid players also direct more ties to their teammates. Similarly, players who have maintained Twitter accounts for a long time attract more following ties, and *time using Twitter* is also predictive of following ties by player *i*. By contrast, the *height* of NBA players and their teammates has little effect on the probability of following ties.

**Fig 4:**
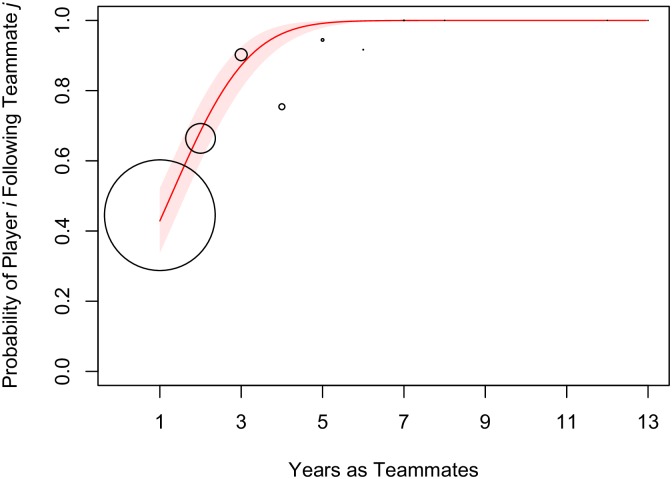
Predicted probability of following ties by experience as teammates. Model predictions are based on simulations from the posterior samples of Model 4. Predictions assume that both player *i* and teammate *j* have not been recognized as All-Star players. All other parameters are held constant at their means or reference values. Points are aggregated and sized proportionally to the number of dyads with a given length of experience as teammates. Shaded intervals show the 90% confidence around model predictions.

The clearest effect in the model is that experience as teammates is highly predictive of following ties on Twitter. As seen in Fig 4, the model predicts that once players have been teammates for several years, they almost invariably follow each other’s accounts. Players who attended the same college also show an increased probability of connecting on Twitter.

By comparing the variances of the random effects, it is evident that the covariates explain a substantial fraction of the variation of teammates as recipients of Twitter ties. That is, the variance of the teammate-level effects declines from 0.71 to 0.50, suggesting that the predictors explain approximately 30% of the variation in attracting followers. By contrast, the other variances increase modestly, and it is evident that the predictor variables in this model account for a minority of the variation in the dataset. Notably, across all models, the reciprocity correlations remain consistently high.

## Discussion

This study indicates that All-Star players on underperforming basketball teams are less likely to follow their teammates’ Twitter accounts than their high-status counterparts on successful teams. In a sports league where elite players are distinguished largely by the success of their respective teams, the status of All-Star players is potentially threatened by their teams’ failures. Although the analysis cannot rule out alternative causal explanations, the tendency for All-Star players on losing teams to distance themselves from teammates is potentially interpretable as a strategic attempt to escape an association with the teams’ poor performance and the broader consequences that ensue from declines in status [11, 17, 56–58].

The moderating effect of team performance does not extend to non-All-Star players, for whom the probability of following teammates on Twitter is generally similar on winning and losing teams. This result is unanticipated given the oft-cited correlation between team cohesion and performance [59]. Among possible explanations, the status of non-All-Star players is seemingly less impacted by their teams’ lack of success, reducing the motivation to distance themselves from teammates. Moreover, relative to their high-status peers, these players may feel a greater obligation to conform to norms or teammates’ expectations about social interactions on Twitter [10]. Overall, for future research on the status of individuals and groups, these results suggest a need to investigate and substantiate the heterogeneous impacts of group performance on the status of individual members [60].

A rationale for using team performance to evaluate and compare elite basketball players is the high task interdependence of basketball relative to other sports, such as baseball [20, 61]. Whereas baseball features a sequential order of individual batters, basketball teams simultaneously coordinate to generate a scoring attempt by one player. Therefore, the statistics of basketball players are more closely linked to the contributions of their teammates whereas it is relatively easier to distinguish the quality and performance of individual baseball players. While team performance is a strong predictor of elite individual status among basketball players, honorific statuses such as enshrinement in the Hall of Fame are less contingent on team success for baseball players. In other words, high task interdependence on teams potentially impedes accurate evaluations of the quality of individual players, which in turn exacerbates the potential for declines in status among individual members of underperforming basketball teams. Hence, for other settings characterized by low task interdependence, such as baseball, the interaction of individual-level status and team performance may be less pronounced [62].

This study uses cumulative All-Star appearances as the measure of players’ status, but alternative measures might merit consideration. In their study of the NBA, for example, Ertug and Castellucci implement a three-year moving window, considering only awards and honors in the previous three seasons as their measure of status [42]. If status is ephemeral, moving windows could be a worthwhile alternative to cumulative measures of individual status. Similarly, the timeframe for measuring team performance in this study was motivated by the advent of Twitter in 2007 and also the assumption that the prolonged success or failure of a basketball franchise establishes its reputation for quality even as the players and personnel change [63, 64]. Given the sample of only 330 players and 30 teams, however, it is likely that alternative measures of individual status and team performance would result in estimated interaction effects that differ in magnitude from the parameters in this analysis.

A consequence of our retrospective measure of team performance is that the analysis does not distinctly reflect the contributions that players have made toward their current team’s performance. That is, regardless of the number of seasons that players have been members of their teams, the statistical models in this study assume that they behave similarly on Twitter as a function of their current team’s weighted prior performance. This assumption may be tenable if players who join inferior teams experience a loss of status that resembles the declines incurred by the members of losing teams. A move to an inferior team may be particularly damaging for high-status players, for whom the transition potentially signals that their anticipated contributions no longer merit contracts from franchises with imminent aspirations of winning a championship. That said, there may be additional dynamics of status, tenure, mobility, and organizational performance that are not identified by the covariates in the models, which explain only a small percentage of the higher-level variation in Twitter ties among teammates.

Methodologically, this study capitalizes on behavioral data, specifically their following ties on Twitter, to examine the social networks of professional athletes. Conventionally, research on team performance and group cohesion has relied on self-reports and psychometric methods that are often infeasible to administer with populations such as professional athletes [27, 65]. Much like email networks and other behavioral data, an additional advantage of Twitter data is that they are unaffected by the biases and demand effects that characterize interview-based methods [66, 67]. Further extensions of this approach could examine the effects of status and group performance on inter-group relationships among members of different teams or organizations [68, 69]. Longitudinal data would permit research on the dynamics of team cohesion and performance, potentially permitting inferences about the causal relationship between those variables. Finally, in addition to studies of the directed network ties, some research questions would benefit from alternative methods, such as content analysis of the language in Twitter messages that are exchanged among team members [70–72].

This study also advances the probit SRM for use by organizational researchers. Whereas researchers have employed the SRM for continuous outcomes in several influential studies [57, 65, 73, 74], analogous studies of binary network ties have tended to rely on a multi-way clustering method developed by Cameron et al. [75]. Unlike the clustering method, the probit SRM advantageously allows the structural dependencies of network data to be explicit and informative, and the coefficients of predictor variables are adjusted to reflect the clustering [76]. Also, the multilevel formulation of the model presented here easily accommodates covariates and imbalanced sample sizes across groups, which pose challenges for conventional ANOVA applications of the SRM [77, 78]. Future extensions of the modeling approach will potentially combine elements of the probit SRM for binary data with longitudinal growth models to better address the temporal dynamics of social networks [79].

## Conclusion

This study provides evidence that relative to their peers, high-status members of underperforming groups associate less with their group members. This result suggests that individuals either embrace or deemphasize their group affiliations in ways that strategically promote their overall status. There are important caveats about generalizing from sports teams to other organizational contexts, but the behavior of NBA players mimics the avoidance and defensiveness that typify management teams during periods of organizational decline [18]. Such avoidance may be particularly likely when the status afforded to individuals is closely tied to organizational performance, as in perceptions of All-Star NBA players [10, 11, 17]. Given evidence that groups fare better when their leaders occupy central positions in intra-group networks [27], the withdrawal of high-status members during periods of poor group performance possibly augurs further declines.

## Supporting information

S1 Fig The number of All-Star players per team as a function of the team’s weighted playoff performance. The line depicts predictions from a Poisson regression model. Shaded intervals show the 90% confidence around model predictions. Points represent the sum of players on each team with experience in at least one All-Star game. Only players who maintained active Twitter accounts were included in the analysis.
(TIF)

S2 Fig Predicted probabilities of following ties by the interactions of team performance and the All-Star status of player *i* and teammate *j*. Model predictions are the means of simulations from the posterior samples of Model 3. All-Star players and teammates are assumed to have 4 All-Star appearances, and non-All-Star players are assumed to have none. Player *i* and teammate *j* are assumed to have played together for one season. All other parameters are held constant at their means or reference values. Depicted points represent the quotients of observed following ties divided by the potential number of ties on each team (i.e., each team’s network density).
(TIF)

S1 Software Probit SRM template for use with Stat-JR software. This template facilitates the analysis of the Social Relations Model for binary, directed ties in multiple groups. It is designed to function with Stat-JR software, available from the Centre for Multilevel Modeling at the University of Bristol.
(PY) Click here for additional data file.

S1 Dataset The dataset, formatted as a comma separated values (csv) file, permits replications and extensions of the analysis. (CSV)
