# Supplementary figures and images for: The effects of individual status and group performance on network ties among teammates in the National Basketball Association
Source: PLoS One. 2018 Apr 30;13(4):e0196013. doi: 10.1371/journal.pone.0196013 (PMC5927430; doi:10.1371/journal.pone.0196013)

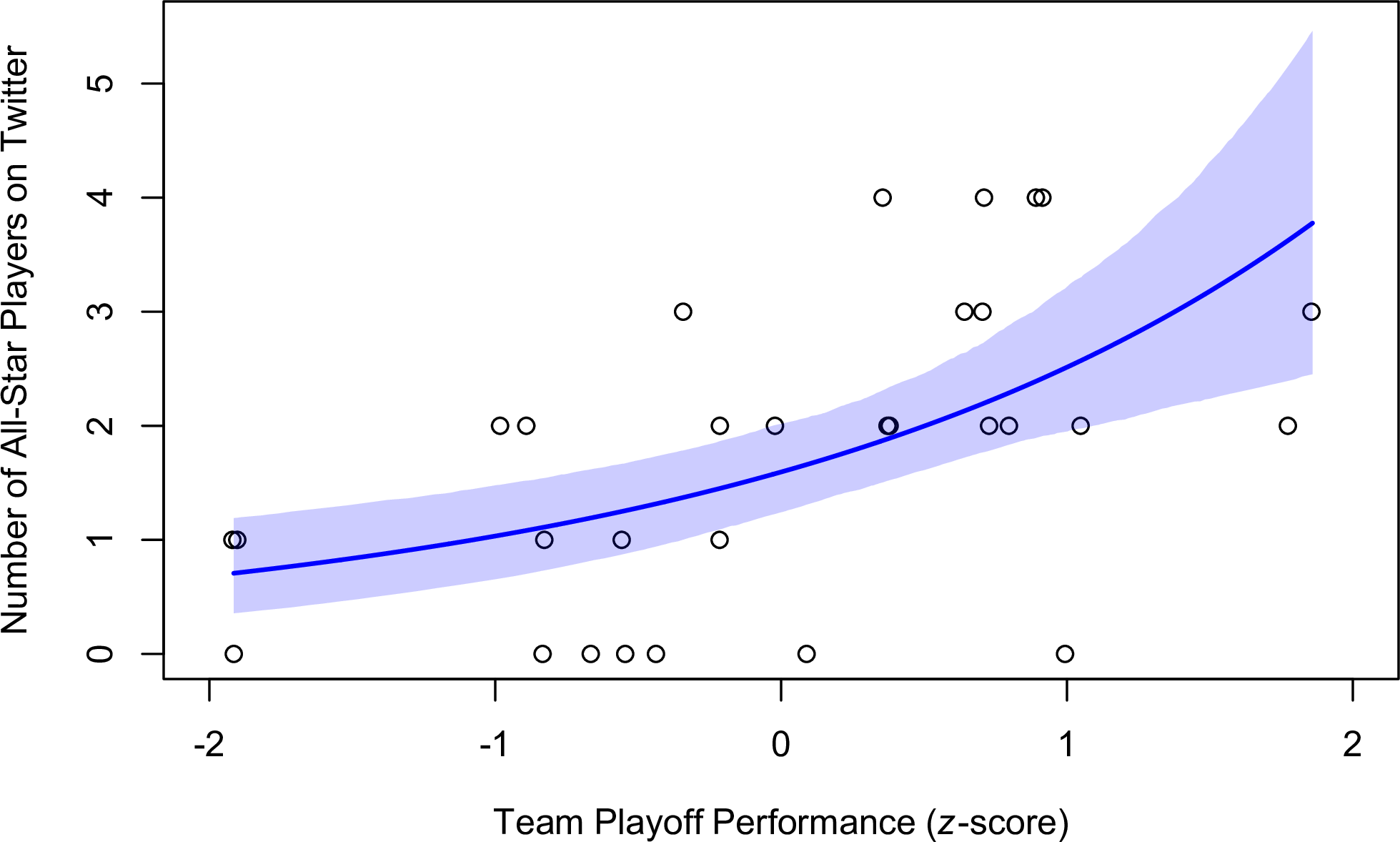

Supplement: S1 Fig — The line depicts predictions from a Poisson regression model. Shaded intervals show the 90% confidence around model predictions. Points represent the sum of players on each team with experience in at least one All-Star game. Only players who maintained active Twitter accounts were included in the analysis. (TIF) [file pone.0196013.s001.tif]

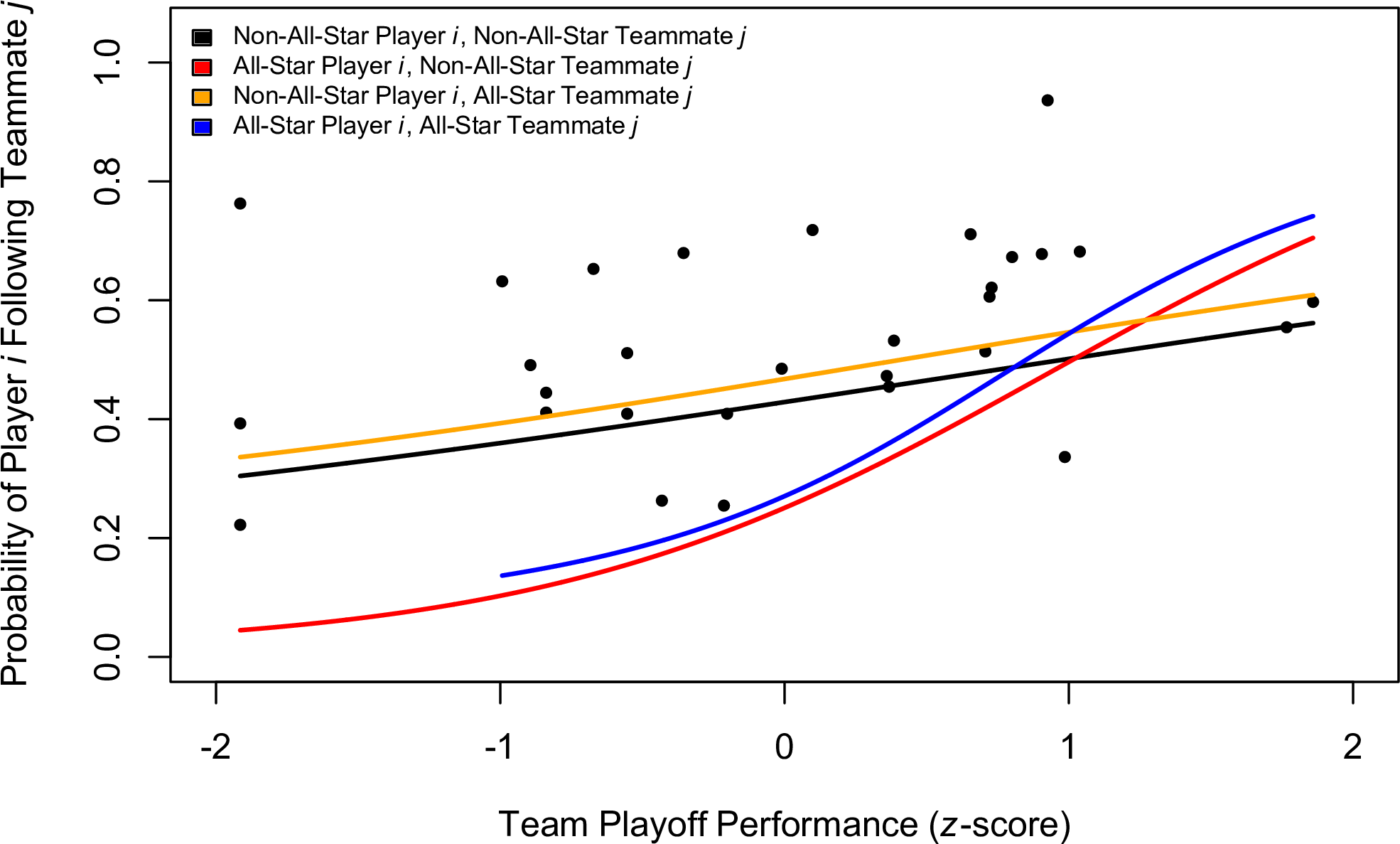

Supplement: S2 Fig — Model predictions are the means of simulations from the posterior samples of Model 3. All-Star players and teammates are assumed to have 4 All-Star appearances, and non-All-Star players are assumed to have none. Player i and teammate j are assumed to have played together for one season. All other parameters are held constant at their means or reference values. Depicted points represent the quotients of observed following ties divided by the potential number of ties on each team (i.e., each team’s network density). (TIF) [file pone.0196013.s002.tif]
